# Modeling and Simulation on Nickel Electroplating Behavior of MLCC with Ni Termination

**DOI:** 10.3390/ma19214722

**Published:** 2026-11-06

**Authors:** Wen-Hsi Lee, Ching Feng Chien

**Affiliations:** Department of Electrical Engineering, National Cheng Kung University, No. 1, University Road, Tainan 701, Taiwan; jeffcian0203@gmail.com

**Keywords:** MLCC, termination, simulation, Ni-plating

## Abstract

This study investigates the influence of electrode geometry, dielectric spacing, and surface treatment on the electroplating behavior of multilayer ceramic capacitors (MLCCs). A series of two- and three-dimensional simulations was performed to analyze potential distribution, electric field strength, current density, and nickel ion concentration under different electrode exposure conditions, including flat, protruded, and recessed configurations. The results indicate that fully exposed and closely spaced electrodes generate stronger electric fields and higher current densities, enabling more efficient and uniform metal deposition. In contrast, recessed or widely spaced electrodes produce weaker fields and limited ion migration, resulting in poor plating performance. Mechanical surface treatment was found to significantly improve electrode exposure and surface uniformity, thereby enhancing plating quality. While the simulations covered a range of structural variations, the experimental portion of this study focuses on a single MLCC design with 2 μm dielectric spacing. Key parameters such as plating duration, mechanical treatment, and chemical surface processing will be further examined to establish correlations with the simulated trends. These findings provide valuable insights for optimizing termination plating processes in advanced MLCC manufacturing.

## 1. Introduction

The multilayer ceramic capacitor (MLCC) is structurally similar to a traditional capacitor but consists of multiple stacked layers. This layered configuration enables the use of extremely thin dielectric layers, thereby achieving high capacitance. The internal electrodes are arranged in an alternating pattern, forming a sandwich structure with the dielectric material [1].

(1) $$C=\frac{\varepsilon \left(\left(N-1\right)A\right)}{d}$$

In Equation (1), Cepresents capacitance, ε is the dielectric permittivity, N is the number of internal electrodes, A is the overlap area, and d is the dielectric thickness. Thus, MLCC performance is strongly dependent on dielectric layer thickness [2]. As MLCCs continue to shrink while achieving higher capacitance, the corresponding reduction in dielectric thickness increases the electric field strength, making these components more susceptible to oxygen-vacancy-related failures. Recent studies further show that as dielectrics become thinner, the size of extrinsic defects becomes comparable to the dielectric spacing, meaning that defects previously considered harmless can now induce catastrophic failure [3]. Traditionally, MLCC termination layers are formed using a dipping process. These terminations typically consist of a copper paste layer, followed by nickel plating and an outer tin layer [4]. The copper layer serves as the initial conductive layer that connects all internal electrodes. Due to its high electrical conductivity, copper ensures a robust electrical connection and strong adhesion to the ceramic body [5]. However, the dipping process can lead to nonuniform layers and requires high curing temperatures, which introduce thermal stress. Over time, this stress can cause microcracks within the termination layer, allowing moisture to penetrate through the copper layer into the MLCC body, ultimately degrading reliability [6]. In conventional MLCCs, the high conductivity of copper electrodes makes the subsequent electroplating process relatively insensitive to plating parameters. In contrast, the innovative technology investigated in this study eliminates the copper terminal electrode entirely. Instead, electroplating is performed directly on the exposed nickel of the internal electrodes. As a result, the density, distribution, and defect conditions of the nickel electrodes have a far greater influence on plating outcomes. Establishing an electroplating model to evaluate these effects offers significant advantages for advancing this copper-free termination technology [7].

Electrodeposition is a widely used method for depositing metallic films and is favored for its simplicity and low energy consumption [8]. A well-established application of electrodeposition is in lithography, where metals are selectively plated onto patterned surfaces [9,10]. The top surface of an MLCC, which contains exposed conductive spots, shares notable similarities with lithographic patterns. This similarity suggests that direct plating onto MLCC surfaces is feasible. Moreover, since the conductive regions consist of the same metal being plated, adhesion can be improved, enabling uniform deposition. This capability opens new opportunities for enhancing MLCC fabrication processes.

However, direct plating also introduces challenges. Removing the traditional dipping process eliminates the cermet layer that typically covers the MLCC surface before plating. This increases hydrogen adsorption and the likelihood of hydrogen diffusion into the ceramic body, potentially reducing reliability [11]. Additionally, the topology of the MLCC surface may be inconsistent: internal electrodes may be partially exposed or recessed, disrupting uniform plating. Previous experiments required copper seeding to stabilize electrical contact during electroplating, though adhesion remained inadequate.

In this study, direct-plated MLCC terminations are analyzed using numerical modeling to identify the key parameters that influence plating behavior. Since electroplating is performed directly on the internal nickel electrodes, their distribution and defect structure play crucial roles in plating performance. Developing a detailed electroplating model, therefore, provides critical insights for implementing copper-free MLCC termination technology [12]. Furthermore, electric field, current density, and ion transport behavior are simulated across various surface topologies to predict the optimal configuration for direct electroplating. A factorial design analysis is also employed to evaluate the consistency between simulation predictions and experimental measurements.

## 2. Modeling Methodology

Understanding the electroplating behavior in multilayer ceramic capacitor (MLCC) terminations requires a comprehensive examination of both the internal and external structural configurations. In this study, a Multiphysics modeling approach was developed to investigate the time-dependent behavior of the system, including electric field formation, potential distribution, current density, and ion migration within the electrolyte. The influence of dielectric spacing, patterned surface design, and electrolyte transport was addressed through a combination of 2-dimensional and 3-dimensional simulations, each tailored to reveal a specific aspect of the plating mechanism [13].

**Figure 1:**
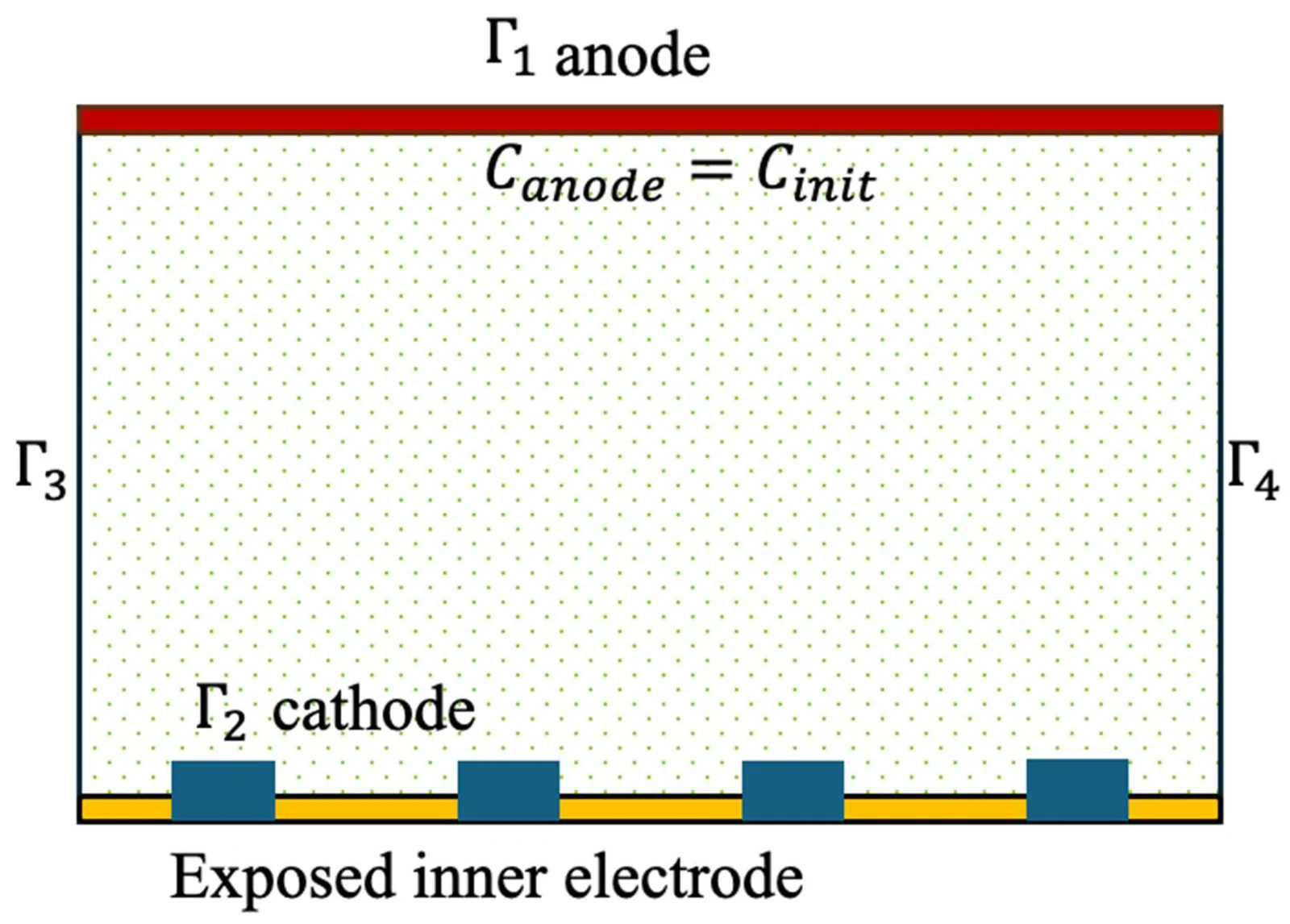
Two-dimensional model to simulate electroplating process.

**Figure 2:**
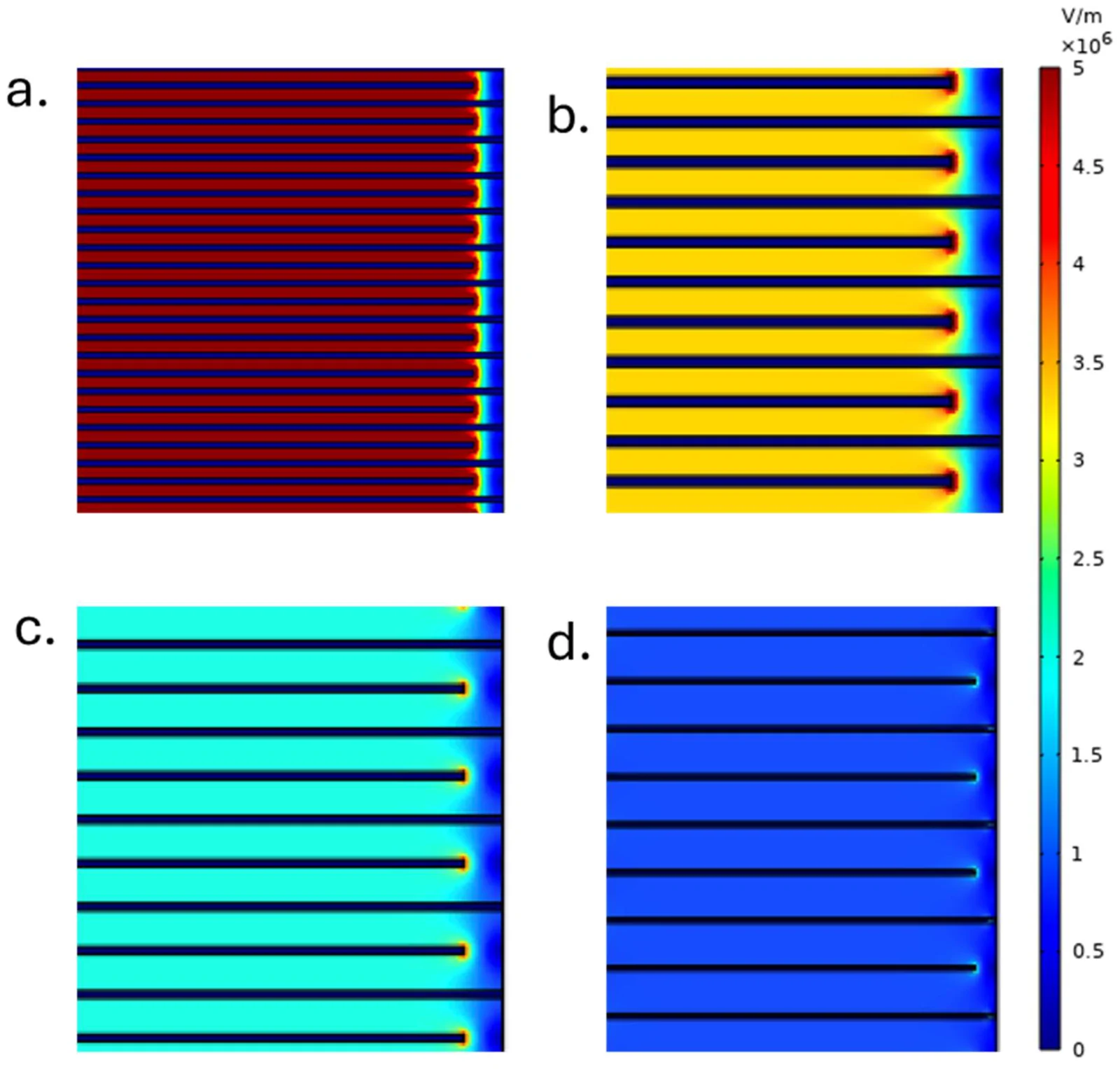
Cross-sectional visualization of volume electric field distribution in MLCC with varying distance between anode and cathode of (**a**) 2 μm, (**b**) 3 μm, (**c**) 5 μm, and (**d**) 10 μm.

The electrochemical potential distribution within the system is governed by both the patterned layout of the surface electrodes and the internal dielectric configuration. A 2D simulation model was constructed to investigate the potential profile along the MLCC termination surface, where the top and bottom boundaries (denoted as Γ_1_ and Γ_2_) serve as the electrochemical interfaces. The electrolyte flows from the left boundary (Γ_3_) to the right boundary (Γ_4_), simulating the flow conditions in a plating bath as illustrated in Figure 1. The electric field for different inner electrodes on MLCC termination when a 5 V working potential is applied, as shown in Figure 2, is calculated using Laplace’s equation [14]. The potential between the electrodes is calculated using Equation (1).

∇^2^*V* = 0 (2)

which, in 2D, the equation expands to

(3) $$\nabla^{2}V=\frac{\delta^{2}V}{\delta^{2}x}+\frac{\delta^{2}V}{\delta^{2}y}$$

The formation and distribution of the electric field are closely governed by the internal dielectric configuration. The distance between adjacent internal electrodes, defined by the dielectric layer thickness, directly determines the field strength and concentration in localized regions. To visualize these effects, analysis through cross-section and the end-surface capturing the electric field behavior across and within the dielectric body during electroplating was performed with Equation (3) [15].

*E* = −∇V (4)

In the electroplating setup, the space between the anode and cathodes is no longer just dielectric but filled with electrolyte. This change significantly influences how the potential and electric field behave. By applying the same equations introduced in Equations (2)–(4), we can calculate the potential distribution within the electrolyte and determine the electric field at every point along the surfaces of the inner electrodes.

Furthermore, the current density in the electrolyte was also the electrolyte’s potential using Ohm’s law.

*i* = −*σ*∇Φ (5)

With the *σ* is the electrolyte conductivity.

At the interface between the cathode–electrolyte interface, the electrochemical reaction rate is calculated by the Butler–Volmer equation [16]. This relationship captures how the local overpotential influences the reaction kinetics.

(6) $$i_{\mathrm{local}}=i_{0}\left[\exp\frac{\left(\alpha_{a}\mathrm{F\eta}\right)}{\mathrm{RT}}-\exp\frac{\left(-\alpha_{c}\mathrm{F\eta}\right)}{\mathrm{RT}}\right]$$

With *i*_0_ is the exchange density, *η* is the overpotential, *α_a_* and *α_c_* are the anodic and cathodic transfer coefficients, and F is Faraday’s constant. The overpotential is calculated from the following:

*η*(*x*,*y*) = *ϕ_inner electrode_* − *ϕ_electrolyte near electrode surface_* *η*(*x*, *y*) = 0 − ϕ(*x*, *y*) *η*(*x*, *y*) = −ϕ(*x*, *y*) (7)

However, in a patterned surface, there is an effect of active area density [8], a, so the current in the area is

(8) $$i_{\mathrm{area}}={a\cdot i}_{0}\left[\exp\frac{\left(\alpha_{a}\mathrm{F\eta}\right)}{\mathrm{RT}}-\exp\frac{\left(-\alpha_{c}\mathrm{F\eta}\right)}{\mathrm{RT}}\right]$$

Furthermore, the deformation of the cathode surface, or the exposed inner electrode, is modeled by Faraday’s law.

(9) $$N_{\mathrm{Ni}}=-\frac{i_{\mathrm{local}}}{2F}$$

The final piece of the analysis involves the distribution of metal ions within the electrolyte during the plating process. As electroplating progresses, ion concentration near the electrode surface decreases, forming a depletion layer due to consumption at the cathode. This ion distribution is governed by Nernst–Planck transport mechanisms [17].

*N_Ni_* = −*D_Ni_*∇*c_Ni_* − *z_Ni_μ_Ni_Fc_Ni_*∇*ϕ_l_* (10)

(11) $$\frac{\delta c_{\mathrm{Ni}}}{\delta t}=\nabla {\cdot N}_{\mathrm{Ni}}=0$$

where N_Ni_ is the nickel ion flux vector, c_Ni_ is the nickel concentration, z_Ni_ is the ion charge, μ_Ni_ is the ion mobility, F is Faraday’s constant, and ϕ is the potential.

**Figure 3:**
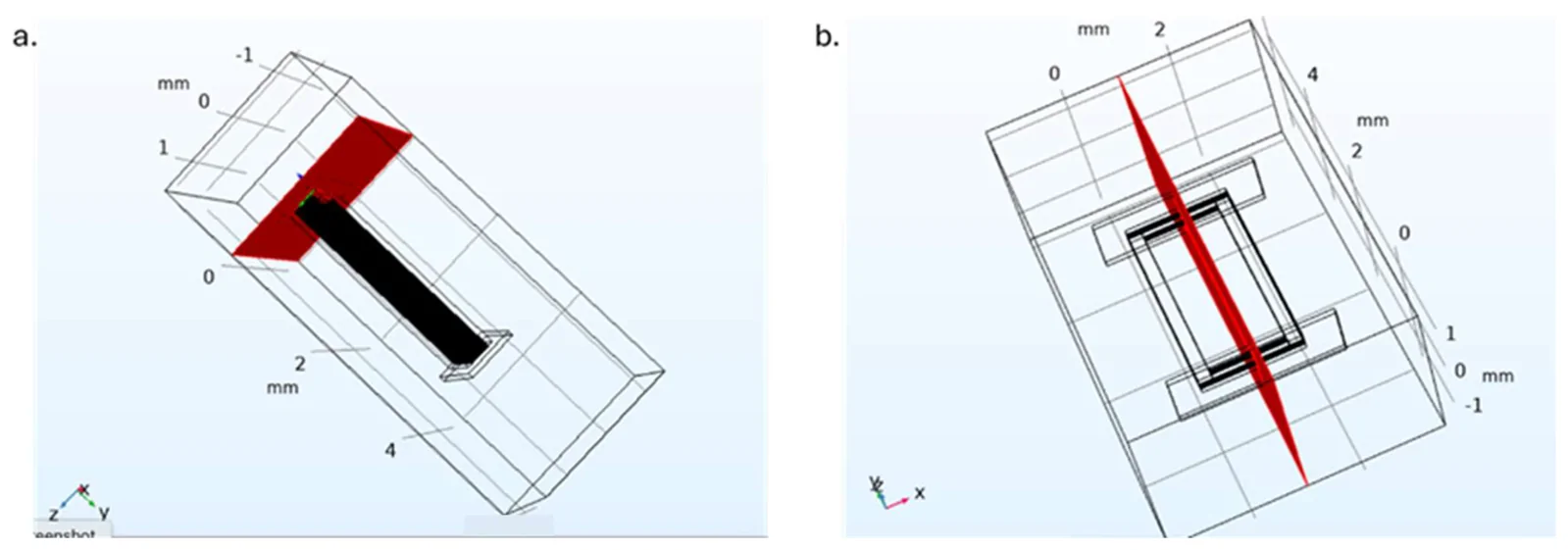
D visualization of an MLCC structure: (**a**) perspective highlighting the electric field intensity along the internal electrode stack on the end-surface, and (**b**) cross-sectional view showing field concentration aligned with the electrode array.

**Figure 4:**
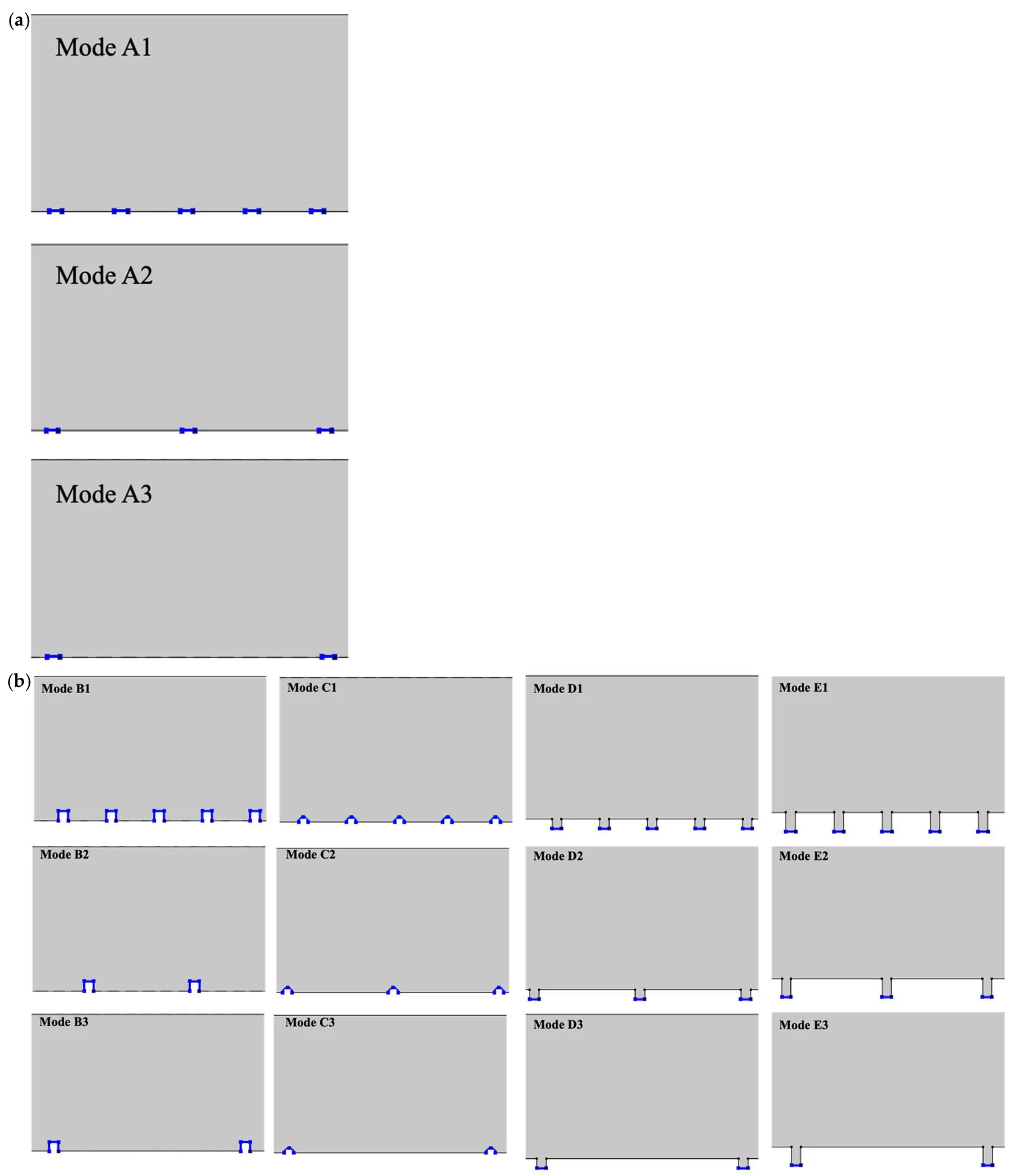
Electrode exposure profiles across plating modes: (**a**) Mode A, which is fully exposed and flat to dielectric surface, and (**b**) Modes B–C protrude from dielectric surface, and Modes D–E are recessed from dielectric surface. Series 1–3 indicate dielectric spacing of 3 μm, 5 μm, and 10 μm between cathodes.

A three-dimensional simulation was performed to examine the electric field distribution at the end surface of an MLCC structure during electroplating. The analysis was carried out from two distinct viewpoints: the end surface and the cross-sectional plane, as depicted in Figure 3. Moreover, a two-dimensional illustration in Figure 4 presents the surface topography, allowing for a more detailed examination of each individual electrode. This perspective plays a key role in revealing how ions move toward the electrode terminations, particularly in multilayer systems with patterned configurations.

## 3. Results

### 3.1. Effect of Dielectric Thickness

A fundamental factor governing the electrochemical performance of multilayer devices lies in their internal architecture—specifically, the dielectric spacing between internal electrodes. This spacing plays a critical role in determining the active area density, defined as the ratio of electrochemically active surface area to the total surface area. When dielectric layers are thinner, adjacent electrodes are positioned more closely, resulting in a higher number of exposed terminations per unit area. This close proximity increases the number of electrochemically active sites, thereby enhancing the active area density. Conversely, thicker dielectric layers increase the separation between electrodes, reducing the density of active regions. Figure 2 illustrates the simulated electric field distribution within an MLCC structure from a cross-sectional perspective. The electric field was evaluated for four different dielectric distances between the anode and cathode: 2 μm, 3 μm, 5 μm, and 10 μm. Although the same external potential of 5 V was applied in all cases, the strength and distribution of the electric field vary significantly due to differences in dielectric thickness. In the 2 μm configuration (Figure 2a), the electric field intensity is the strongest, approaching values close to 5 × 10^6^ V/m (shown in red). The field lines are dense and tightly confined, demonstrating that a shorter electrode gap produces a more concentrated and stronger electric field. Such conditions promote efficient ion migration, which is beneficial for electrochemical reactions such as electroplating. When the spacing increases to 3 μm (Figure 2b), the field intensity becomes slightly weaker, shifting from deep red to yellow. Although the field remains strong, it spreads more vertically, indicating the early onset of field dissipation across the dielectric. At 5 μm (Figure 2c), the electric field becomes considerably weaker and more diffuse. Cyan and green colors dominate, signifying moderate field strength. This diluted field reduces ion acceleration, potentially slowing the electroplating process [18]. With a 10 μm spacing (Figure 2d), the field strength drops sharply, shown in blue. The field is weak and extended over a larger volume, providing greater resistance to ion migration. The increased distance suppresses the electric driving force that propels ions through the electrolyte. Overall, these simulations confirm that smaller dielectric spacing produces stronger, more localized electric fields, while larger gaps diminish field intensity and electrochemical effectiveness. This insight is critical for MLCC design, particularly in electroplating processes where electric field strength directly influences deposition rate and uniformity.

**Figure 5:**
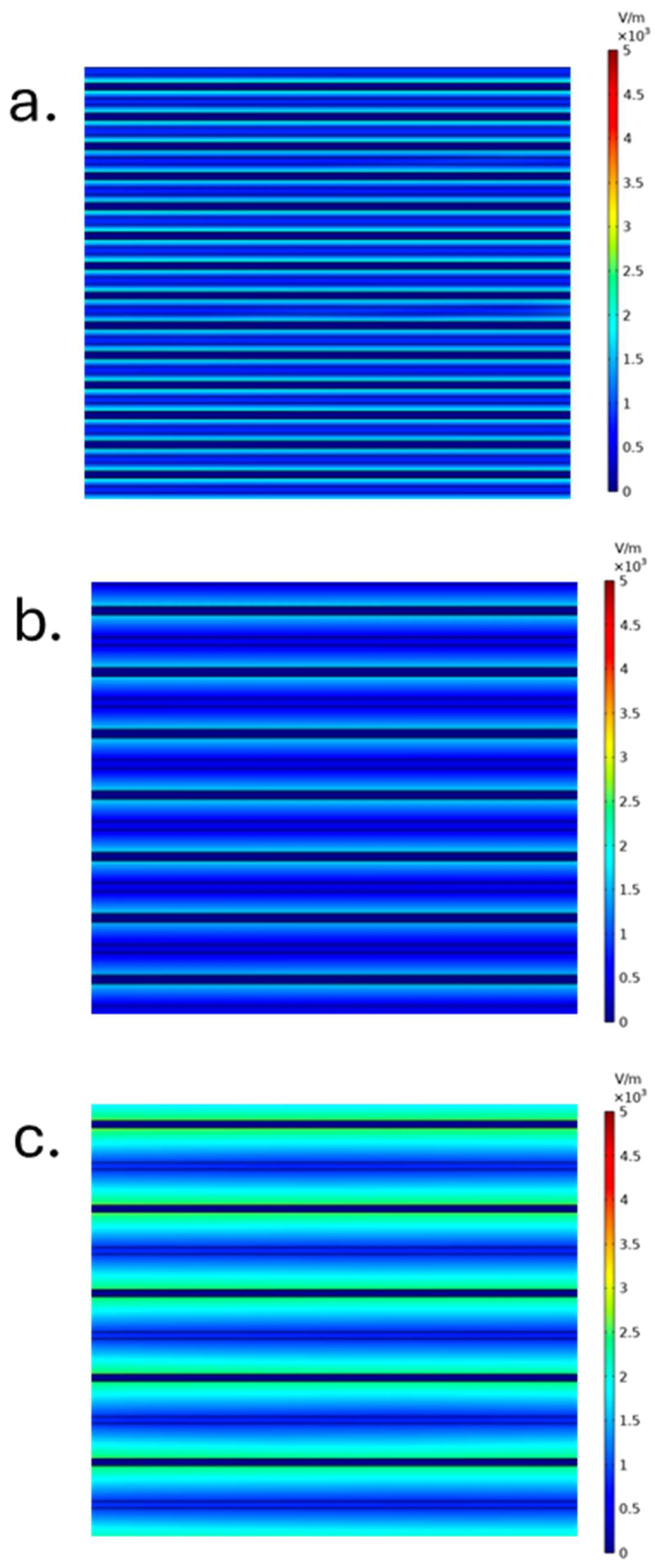
Electric field magnitude distribution (V/m) in MLCC viewed from surface of end-surface perspective of MLCC with dielectric distances of (**a**) 3 μm, (**b**) 5 μm, and (**c**) 10 μm.

**Figure 6:**
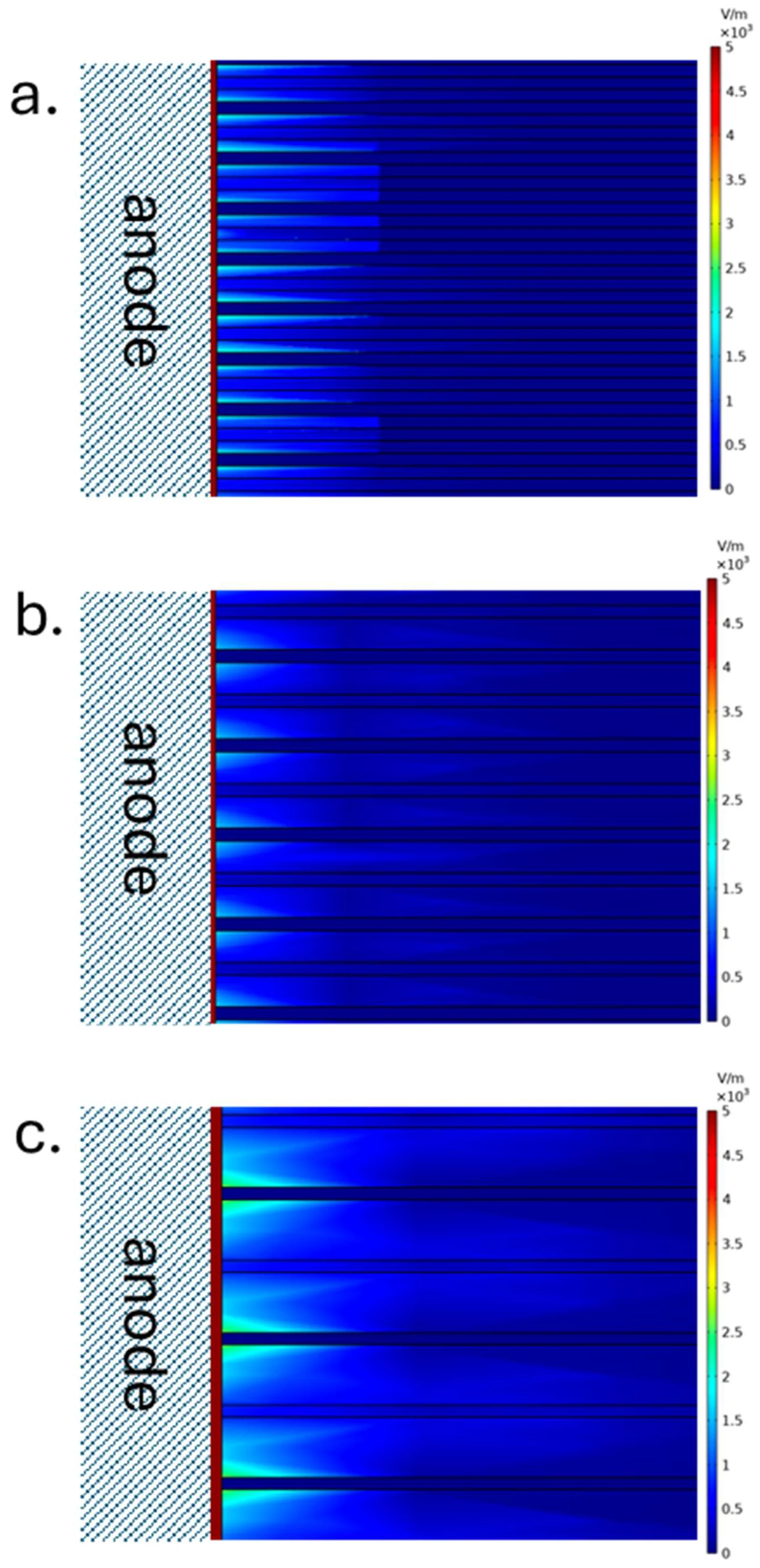
Electric field magnitude distribution (V/m) in MLCC viewed from a cross-sectional perspective of MLCC with dielectric distances of (**a**) 3 μm, (**b**) 5 μm and (**c**) 10 μm.

**Figure 7:**
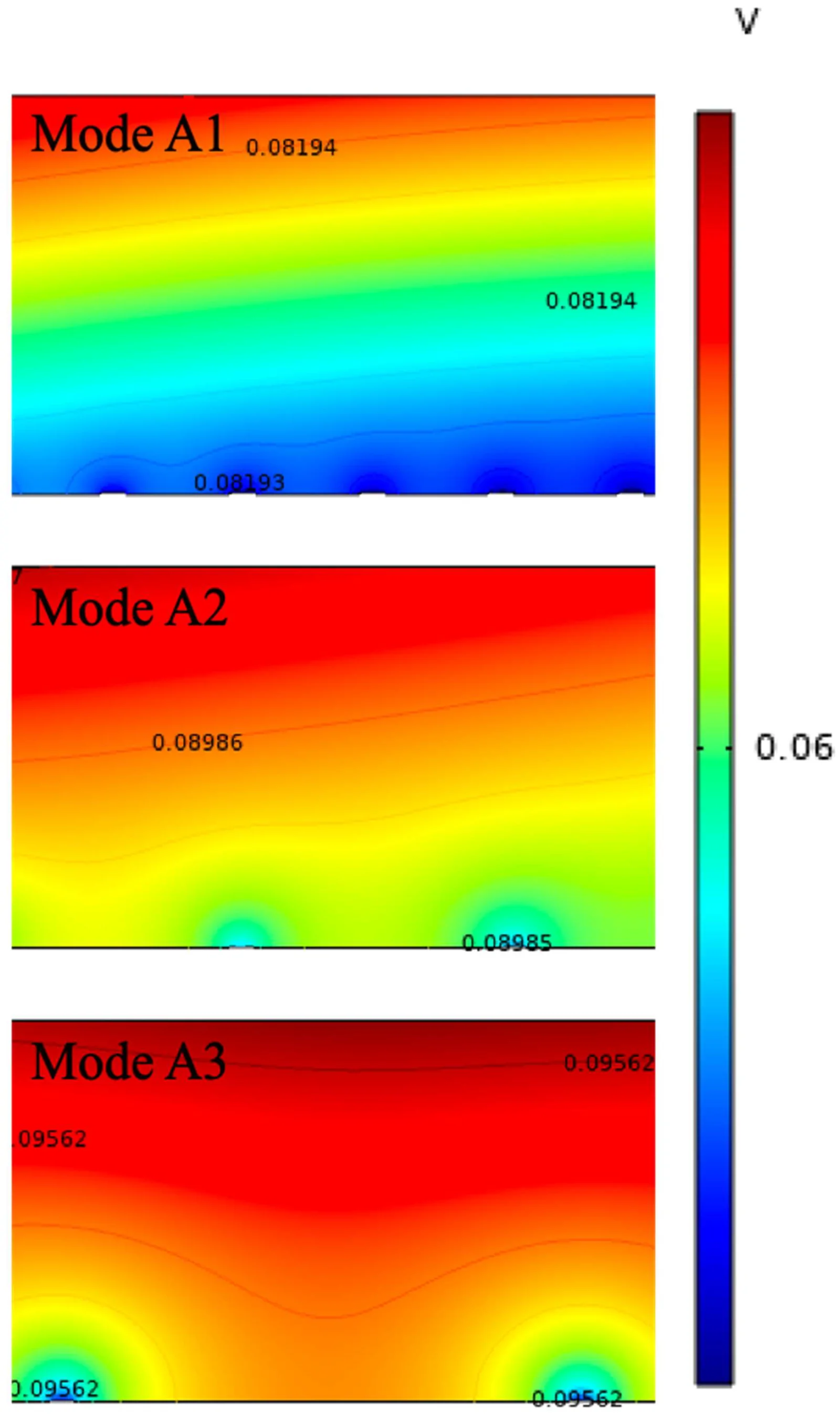
Simulated potential distribution for Mode A, featuring fully exposed electrodes flush with the dielectric surface. Series 1–3 represent dielectric spacings between cathodes of 3 μm, 5 μm, and 10 μm.

A three-dimensional simulation (Figure 3) further analyzes the electric field behavior on the end surface of an MLCC during electroplating under a 5 V anodic bias. The resulting electric field distributions are shown in Figure 5 and Figure 6. In the MLCC with the narrowest dielectric gap (2 μm), the field distribution appears smooth and uniformly moderate. Deep blue dominates the color scale, corresponding to surface field values below 1.5 × 10^3^ V/m. The fields are evenly spaced with minimal lateral leakage, suggesting strong confinement between electrodes and reduced surface-level interference. At 3 μm spacing, the surface field intensity increases slightly. The bands become thicker and brighter, with light blue to cyan regions reaching approximately 2 × 10^3^ V/m. Fringe effects begin to appear near electrode edges. For 5 μm spacing, surface field distortion becomes more pronounced. Cyan regions intensify around electrode edges, indicating stronger fringe field formation and localized hotspots. At the widest spacing of 10 μm, the surface field increases further, with cyan and light green shades dominating and some regions exceeding 3 × 10^3^ V/m. The greater spacing reduces field confinement and allows more lateral spread. A more detailed two-dimensional analysis of the surface (Figure 7) was performed at an applied potential of 0.1 V. Although the applied voltage is identical across configurations, variations in electrode geometry and spacing cause notable differences in internal potential distribution. In Mode A1 (4 μm spacing), the potential near the cathode remains low and uniform at approximately 0.08194 V. The potential gradient is smooth, indicating a direct electric field path and minimal distortion, which supports efficient ion transport. In Mode A2 (10 μm spacing), the cathode potential increases to about 0.08986 V, and the potential lines become slightly curved. The wider spacing introduces mild field non-uniformity and reduces ion mobility in certain regions. In Mode A3 (20 μm spacing), the cathode potential rises further to ~0.09562 V, and the potential gradient becomes less steep. The increased dielectric gap weakens the electric field and disperses its distribution, reducing ion migration effectiveness [19].

**Figure 8:**
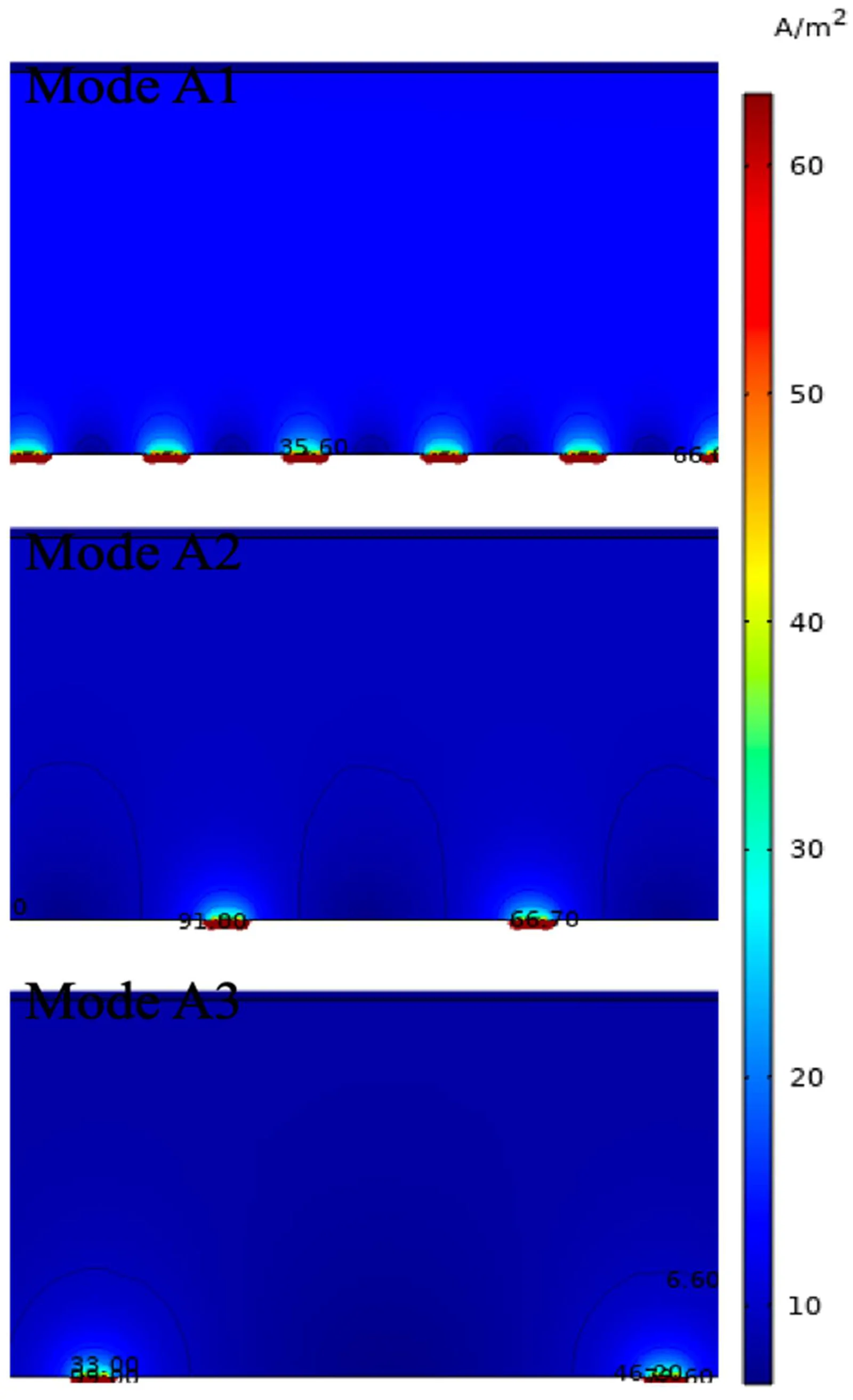
Simulated current density for Mode A, featuring fully exposed electrodes flush with the dielectric surface. Series 1–3 represent dielectric spacings between cathodes of 3 μm, 5 μm, and 10 μm.

Figure 8 shows the simulated current density distributions for Mode A (flat, fully exposed electrodes) with dielectric spacings of 3 μm, 5 μm, and 10 μm. The spacing between end electrodes corresponds to twice the dielectric thickness plus the electrode thickness. In Mode A1 (3 μm), the current density reaches its highest values, exceeding 60 A/m^2^. The current spreads uniformly along the bottom interface, forming a continuous conductive region. Strong field overlap and efficient ion transport lead to enhanced plating activity. In Mode A2 (5 μm), the current density remains high but forms more localized peaks. Although hotspots above 50 A/m^2^ still appear, the increased spacing reduces field overlap and creates more isolated current pathways. In Mode A3 (10 μm), the current density decreases significantly. Peak values drop to ~35–45 A/m^2^ and occur only in localized regions, with minimal current between electrodes. The increased ion migration distance reduces electroplating efficiency. Thus, reducing dielectric spacing intensifies current density and improves plating uniformity, while larger spacing weakens field strength and leads to dispersed, less efficient current distribution. This highlights the importance of small cathode spacing for achieving dense and uniform metal deposition in MLCC terminations.

**Figure 9:**
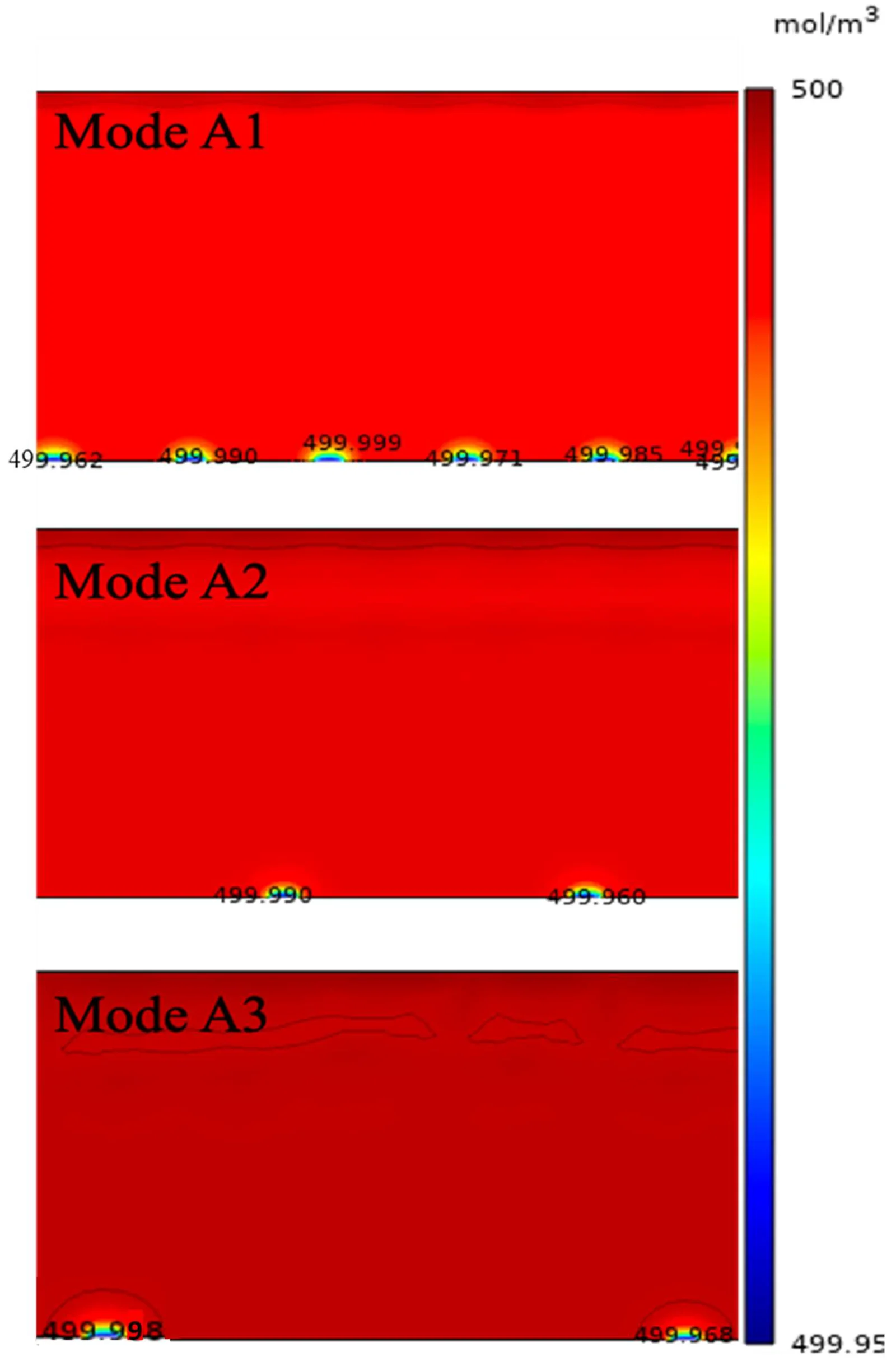
Simulated nickel ion distribution for Mode A, featuring fully exposed electrodes flush with the dielectric surface. Series 1–3 represent dielectric spacings between cathodes of 3 μm, 5 μm, and 10 μm.

Figure 9 presents the simulated nickel ion concentration distribution for Mode A configurations. In Mode A1, nickel ion concentration shows only slight depletion near the electrode surfaces, staying near the bulk value of 500 mol/m^3^. Minor dips appear near electrode tips due to active ion consumption, but efficient bulk replenishment prevents significant depletion. In Mode A2, depletion zones become more pronounced, and ion recovery requires a longer vertical distance. Wider electrode spacing reduces ion transport efficiency. In Mode A3, depletion is more severe and widespread, indicating ion starvation and reduced plating efficiency. Increased spacing limits ion replenishment at the cathode. Overall, narrower cathode spacing improves ion accessibility and maintains higher local concentrations during electroplating, whereas wider gaps hinder ion delivery and reduce deposition performance.

### 3.2. Effect of Surface Topography

Beyond internal electrode architecture, the surface topography of the electrode plays a crucial role in determining electrochemical activity. In patterned electrodes, only specific regions of the surface actively participate in electrochemical reactions, making it essential to distinguish between geometric and electrochemically accessible areas. Although the geometric area may appear large, only the patterned exposed regions contribute to electrochemical functionality, especially in high-resolution electrode designs.

Figure 4b illustrates the electrode surface configurations and the distribution of active regions. A simulation model was constructed to investigate electroplating behavior on these surfaces, including potential distribution, ion concentration, and current density evolution [20].

**Figure 10:**
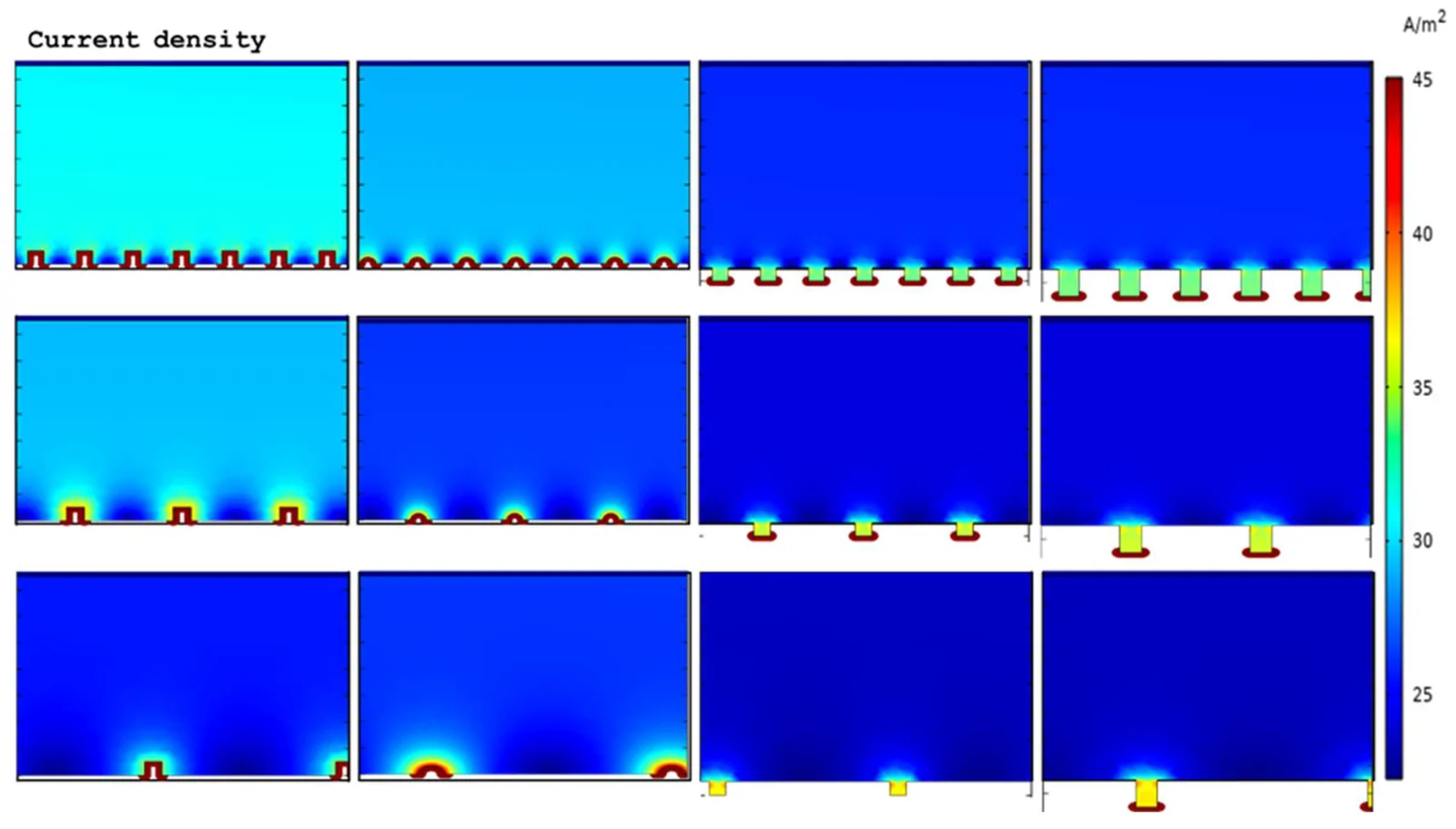
Simulated current density for electrode exposure profiles across plating modes. Modes B–C feature protruded electrodes above the dielectric surface, while Modes D–E contain recessed electrodes below the dielectric. Series 1–3 correspond to dielectric spacings of 3 μm, 5 μm, and 10 μm between cathodes.

Figure 10 presents the simulated current density distributions across different electrode exposure profiles (Modes B, C, D, and E), each with three dielectric spacings (3 μm, 5 μm, and 10 μm). Current density is shown in A/m^2^, highlighting how electrode geometry and spacing influence electrochemical activity. As described earlier, Mode A—with flat, fully exposed electrodes—exhibits the highest current density. The strong and uniformly distributed red regions indicate fast and uniform plating, making Mode A the most efficient configuration. In the B-series (protruded square electrodes), Mode B1 shows the highest current density, exceeding 26 A/m^2^. As spacing increases (Modes B2 and B3), current density decreases, with Mode B3 showing the lowest values. A notable feature across the B-series is the strong field crowding at electrode corners, which produces intense localized red regions. In the C-series (protruded semicircular electrodes), a similar trend is observed, although overall current densities are lower. In Mode C1, the field concentrates at the topmost point of the curved electrode, while Modes C2 and C3 show decreased plating performance and current densities below 10 A/m^2^. The rounded geometry distributes the electric field more smoothly than in Mode B but still exhibits pronounced peaks at curved tips. In the D-series (electrodes recessed by 1 μm), current density drops substantially, with blue regions dominating. Field penetration through the dielectric is limited, resulting in current densities below 15 A/m^2^. Increasing dielectric spacing further weakens plating performance, with Mode D3 performing the worst. In the E-series (electrodes recessed by 2 μm), current densities drop even further. Modes E2 and E3 show values below 5 A/m^2^, and electric field suppression is severe, making these configurations the least effective. Across these modes, protruded electrodes (B and C) clearly perform better than recessed designs (D and E). Within the protruded group, square-shaped electrodes (B-series) outperform semicircular ones (C-series) due to stronger field concentration at sharp corners. Field crowding phenomena create localized peaks in both geometries, accelerating plating at specific points. As the dielectric spacing increases, the electric field overlap diminishes. Reduced field interaction lowers current density and decreases plating rate uniformity across all electrode designs. In electroplating, Ni^2+^ ions migrate toward the cathode via diffusion, migration, and convection, though the latter is negligible here. Ion depletion occurs when ions are consumed faster than they can be replenished from the bulk electrolyte.

**Figure 11:**
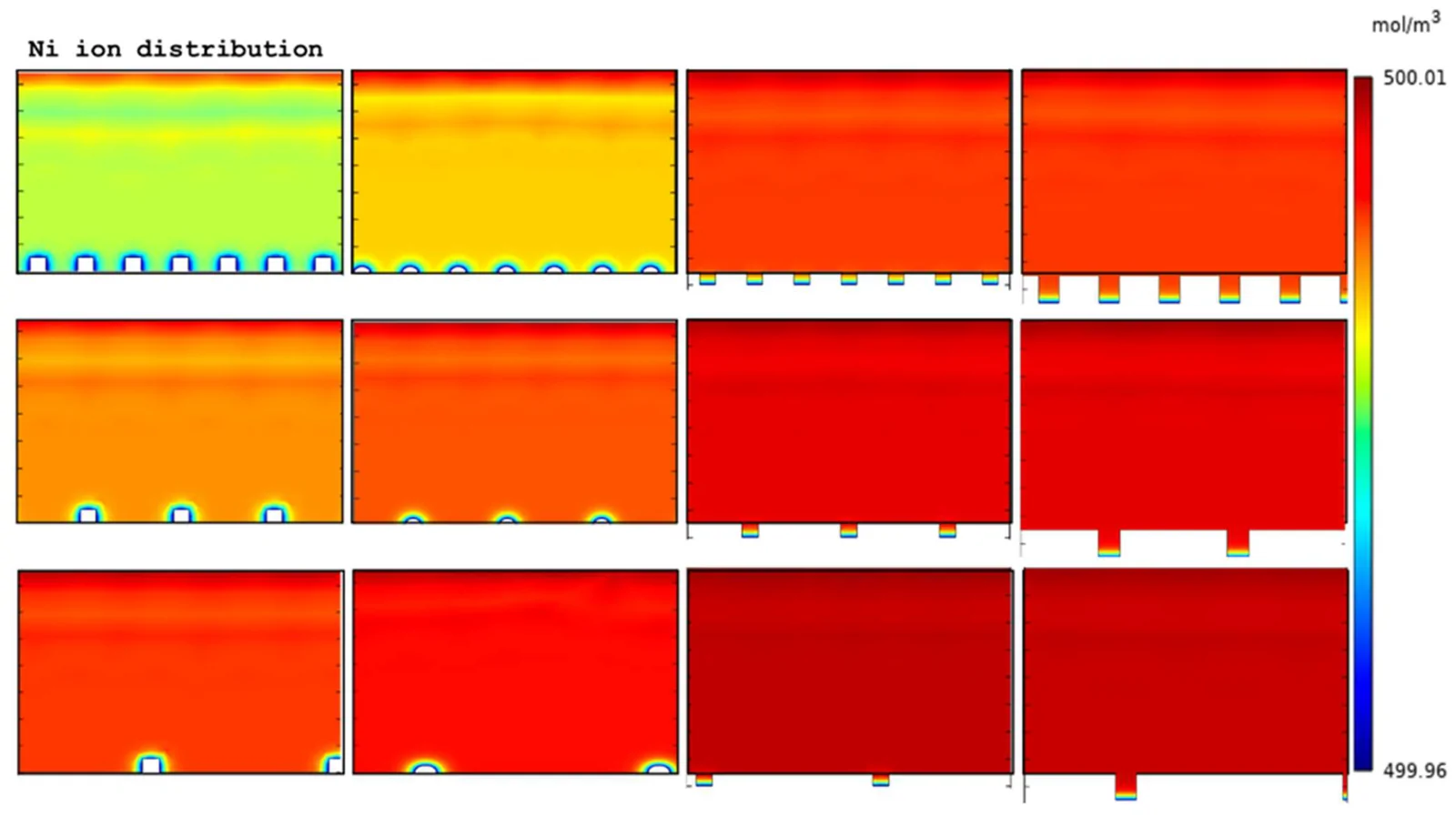
Simulated Ni ion density for electrode exposure profiles across plating modes. Modes B–C feature protruded electrodes above the dielectric surface, while Modes D–E contain recessed electrodes below the dielectric. Series 1–3 correspond to dielectric spacings of 3 μm, 5 μm, and 10 μm between cathodes.

Figure 11 shows nickel ion concentration distributions for Modes B, C, D, and E. In the B-series, Mode B1 shows the strongest depletion near electrode tips, indicating high plating activity. As spacing increases (Modes B2 and B3), depletion becomes weaker and more localized. The C-series exhibits similar but slightly weaker depletion patterns. Mode C1 shows noticeable ion consumption, but the affected zone is narrower compared to Mode B1. Increasing spacing again reduces depletion and plating activity. In contrast, the recessed modes (D and E) show almost no depletion. Ion concentration remains near 500 mol/m^3^ even near the electrode surfaces, indicating extremely limited electrochemical activity due to restricted field penetration. These results confirm that protruded electrodes with narrow spacing provide the most active and efficient electroplating conditions. Sharper geometries (Mode B) create stronger field concentration and more aggressive ion draw than curved ones (Mode C). Recessed electrodes (Modes D and E), especially with larger spacing, perform poorly due to limited field access and minimal ion migration. A summary of the simulation results for potential distribution, current density, and nickel ion concentration across all MLCC termination models is presented in Table 1.

### 3.3. Electroplating Validation

**Figure 12:**
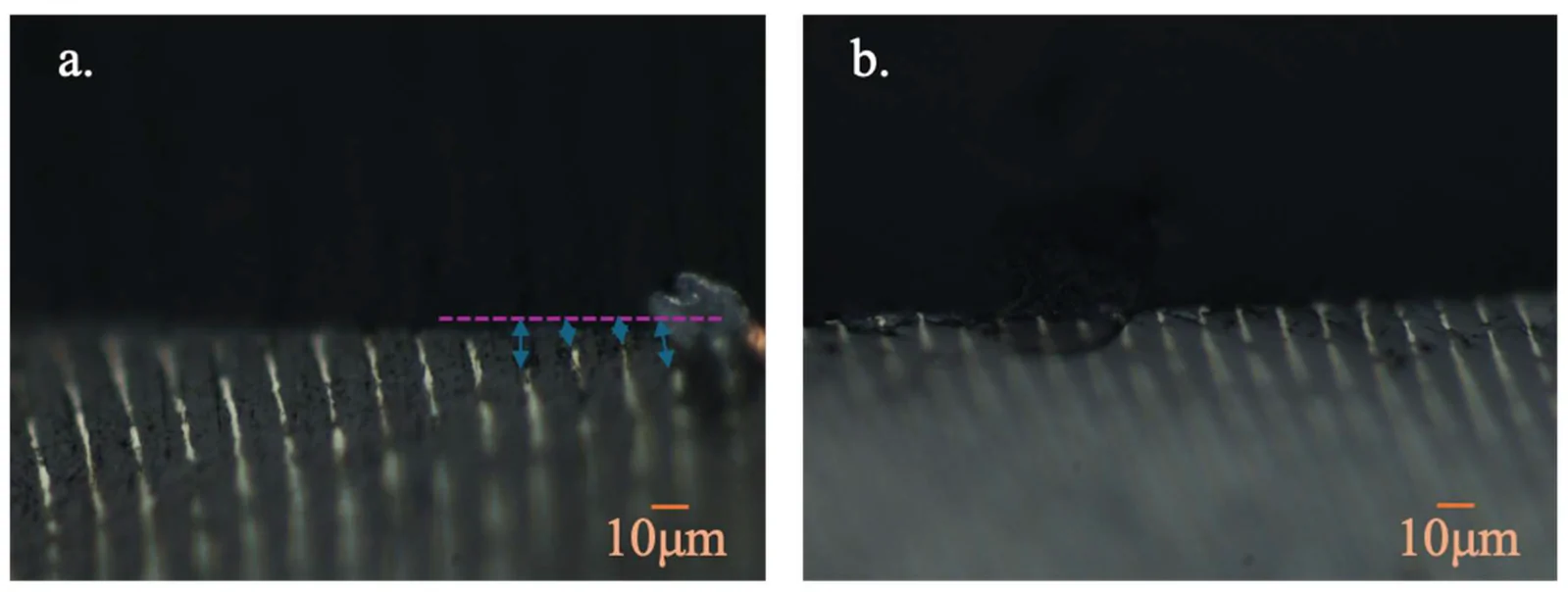
Cross-section of MLCC body (**a**) without mechanical surface treatment and (**b**) with mechanical surface treatment.

**Figure 13:**
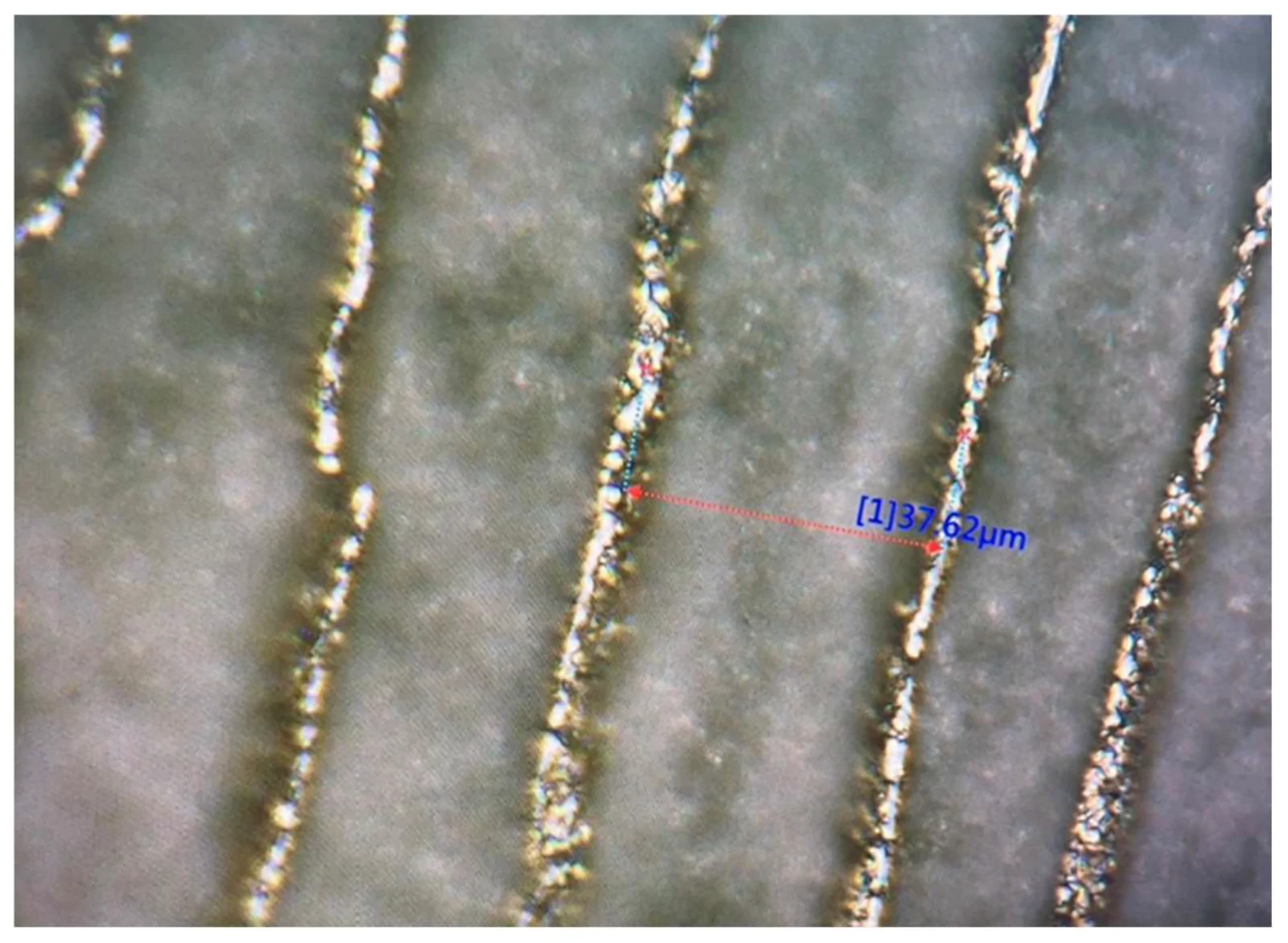
The surface of MLCC with dielectric distance of 20 μm with nickel deposited on top of the electrodes.

The simulation modes are designed based on the surface topography of MLCCs. Figure 12 shows the MLCCs’ cross-section. The MLCC that has not undergone mechanical surface treatment typically exhibits a rougher surface, where some electrodes appear recessed at varying depths. In contrast, those that have been mechanically treated display more exposed electrodes and a flatter, more uniform surface profile. While the dielectric spacing primarily influences the electroplating capability, as shown in Figure 13, an excessively large spacing can hinder plating coverage and make it difficult to electrically connect one electrode to another. As a result, simulations suggest that larger gaps lead to stronger local electric fields. Nevertheless, for this experimental study, we focused on a single type of MLCC with a dielectric spacing of two microns. The parameters under investigation include plating duration, the presence or absence of mechanical surface treatment, and the application of chemical surface treatment.

To investigate the surface variables of electroplating on the termination coverage, a two-level full factorial design DOE approach was used. This would reduce the number of experiments to be performed (and hence costs), allow identification of combinations of variables that would produce a required (not necessarily maximum) coating thickness, and permit an evaluation of the interaction of variables [21].

In the modeling simulation in the earlier section, we know the dielectric distance was one of the factors of the electrodeposition process. In this section, we want to see the effect of the surface condition, whether the protruding or receding electrodes affect the result. The factor of pickling or acid bath is also included to see if the cleanliness of the surface will influence the result. So, in this experiment, we put the factors as plating duration, mechanical surface treatment time, and pickling process, as seen in Table 2. The analysis was using a general factorial with a factor mechanical surface treatment time (A), which has three levels (0, 1, 2), the pickling process (B), which has two levels (yes and no), and plating duration (C) has two levels (40 min, 50 min). This experiment run is 12. Factorial design allows an investigation of the interaction of factors on the result as the coverage. For two-way interactions, there will be AB, AC, and BC combinations. And for three-way interactions, there will be ABC combinations.

The regression equation is

$$\begin{array}{cc} \text{Ni coverage} & =\;66.784\;-62.95\;A_{0}+29.97\;A_{1}+32.99\;A_{2}\\ & -\;1.566\;B_{0}+1.566\;B_{1}-0.763\;C_{40}+0.763\;C_{50}\\ & -\;2.26\;A_{0}\times B_{0}+2.26\;A_{0}\times B_{1}+0.64\;A_{1}\times B_{0}\\ & -\;0.64\;A_{1}\times B_{1}+1.62\;A_{2}\times B_{0}-1.62\;A_{2}\times B_{1}\\ & -\;1.52\;A_{0}\times C_{40}+1.52\;A_{0}\times C_{50}+0.73\;A_{1}\times C_{40}\\ & -\;0.73\;A_{1}\times C_{50}+0.79\;A_{2}\times C_{40}-0.79\;A_{2}\times C_{50}\\ & +\;0.815\;B_{0}\times C_{50}-0.815\;B_{0}\times C_{50}-0.815\;B_{1}\times C_{40}\\ & +\;0.815\;B_{1}\times C_{50} \end{array}$$

**Figure 14:**
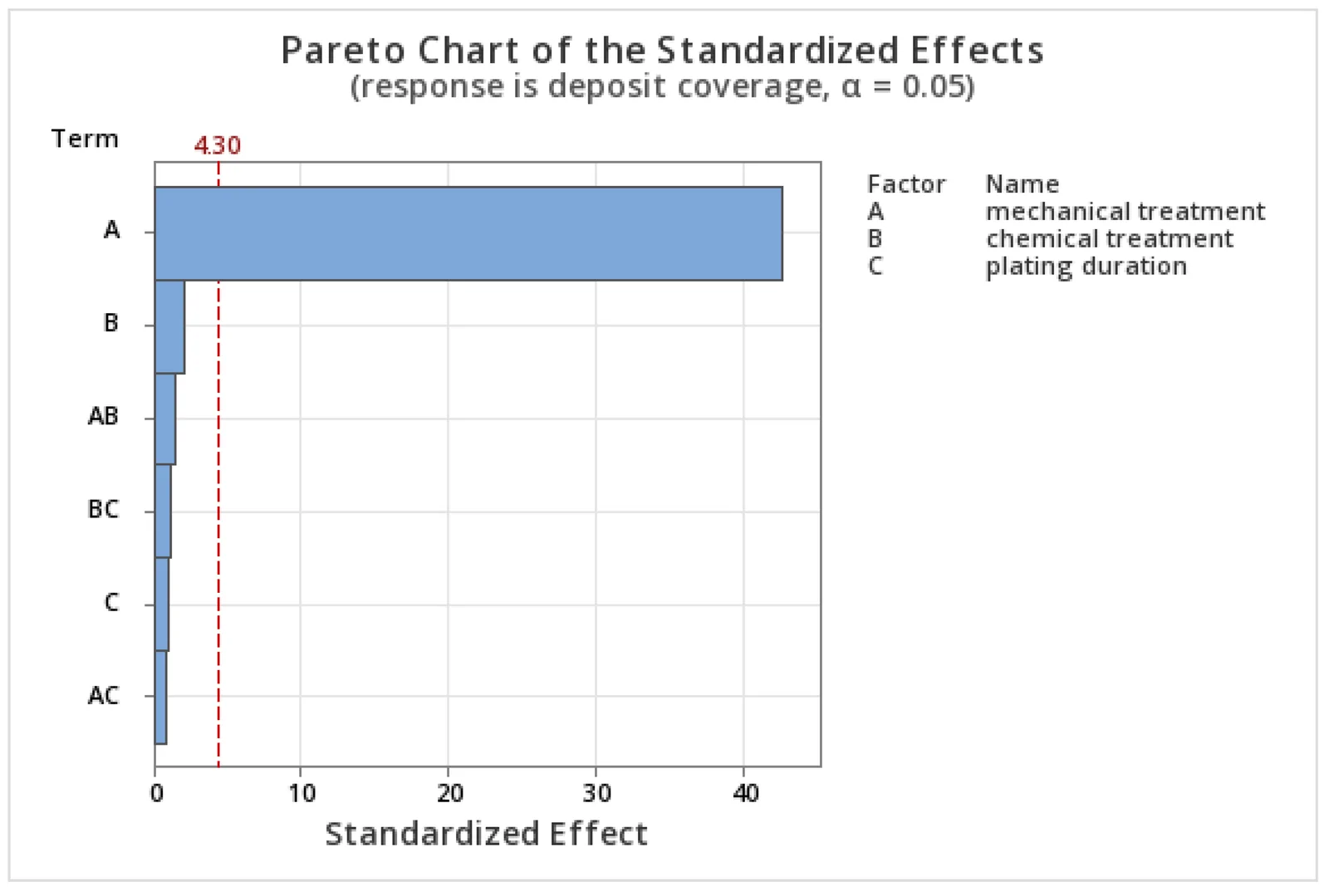
Pareto chart from the design of experiment, a general factorial design to determine the key factor required in the electroplating process. The tumbling time is the mechanical surface treatment that is needed during the sample preparation.

Figure 14 presents one of the key outputs from the DOE analysis: the Pareto chart. In this chart, the horizontal bars that extend beyond the vertical dotted significance threshold correspond to statistically significant factors. According to the analysis of variance (ANOVA), the mechanical surface treatment factor shows a very low *p*-value of 0.001. Since significance is determined by a *p*-value below 0.025, this confirms that mechanical treatment has a strong influence on plating performance. This observation is consistent with the simulation results, in which protruded electrodes—achieved through mechanical surface treatment—significantly enhance electroplating efficiency. While electroplating theories generally assume idealized conditions—such as uniform electric fields, constant ion concentrations, and perfectly planar electrodes—industrial environments deviate substantially from these assumptions. In theory, current distribution and deposition rates can be predicted based on electrochemical kinetics and mass transport models. However, in practice, factors such as anode placement, electrode geometry, electrolyte agitation, and localized concentration gradients introduce non-uniformities that limit the direct applicability of theoretical models.

In real plating systems, electrolyte flow and ion replenishment are influenced by equipment design, temperature fluctuations, and impurities that are difficult to fully capture in simulations. For example, in industrial plating baths, the distance and orientation between the anode and the cathode significantly affect the local current density, often resulting in thickness variations or edge-enhancement effects that are not fully predicted by idealized models. Nevertheless, theoretical simulations remain valuable for understanding the fundamental electrochemical behavior of direct nickel plating on MLCC terminations.

## 4. Conclusions

This study demonstrates that electrode geometry, surface treatment, and dielectric spacing all play critical roles in determining electroplating behavior in MLCC structures. Among the various electrode configurations examined, fully exposed and closely spaced cathodes produce the most efficient plating results, characterized by high and uniform current density, strong electric fields, and active nickel ion migration. Mechanical surface treatment is shown to be particularly important, as it increases electrode exposure and smooths the surface, thereby improving plating uniformity. Although dielectric spacing does not directly determine whether electroplating can occur, it critically influences the local electric field strength and thus governs ion transport efficiency. Excessively large spacing weakens field interaction, leading to reduced plating coverage and lower deposition effectiveness.

## Figures and Tables

**Table 1:** Summary of simulated electrode configurations (Modes A–E) for topographical analysis of nickel electroplating on varied surface geometries.

Mode	Electrode Shape	Position Relative to Surface
A	Flat	Flush with dielectric surface
B	Square Protrusion	Raised by 1 µm
C	Semicircle Protrusion	Raised by 1 µm
D	Square Recessed	Sunken by 1 µm
E	Square Recessed	Sunken by 2 µm

**Table 2:** ANOVA of design of experiment using general factorial for the coverage level of nickel on MLCC termination using direct electroplating process.

Source	DF	Adj SS	Adj MS	F-Value	*p*-Value
Model	9	23,885.9	2654	408.71	0.002
Linear	4	23,831.4	5957.8	917.5	0.001
mechanical surface treatment	2	23,795	11,897.5	1832.21	0.001
pickling	1	29.4	29.4	4.53	0.167
time	1	7	7	1.08	0.409
2-Way Interactions	5	54.5	10.9	1.68	0.414
mechanical surface treatment pickling	2	32.7	16.3	2.51	0.285
mechanical surface treatment time	2	13.8	6.9	1.07	0.484
Pickling time	1	8	8	1.23	0.384
Error	2	13	6.5		
Total	11	23,898.8			

## Data Availability

The original contributions presented in this study are included in the article. Further inquiries can be directed to the corresponding author.
